# *Gigantochloa falcihumeris* (Poaceae, Bambusoideae), a New Paleotropical Woody Bamboo Species From Southwest Yunnan, China

**DOI:** 10.1002/ece3.72849

**Published:** 2026-03-09

**Authors:** Jian‐Wei Li, Chao‐Mao Hui, Wei‐Yi Liu, Mao‐Sheng Sun, Wan‐ling Qin, Hao‐Feng Bao, Ru‐li Zhang

**Affiliations:** ^1^ Institute of Bamboo and Rattan, Sympodium Bamboo Engineering Technology Research Center, College of Forestry Southwest Forestry University Kunming Yunnan China; ^2^ Guangnan Babao Provincial Nature Reserve Administration Bureau Wenshan Yunnan China; ^3^ Forestry Bureau of Nayong County Bijie Yunnan China

**Keywords:** *Gigantochloa*, phylogenetic tree, taxonomy, woody bamboo, Yunnan

## Abstract

A new species *Gigantochloa* is described and illustrated from Southwest Yunnan, China. The new species is similar to *G. verticillata* and *G. felix* in general appearance, but differs by its taller culms, internodes without stripes, culm leaf sheath shoulders prominent upward forming ca. 1 cm long falcate appendage, culm blade erect, culm leaf auricle absent, and culm leaf ligule ca. 5 mm tall. Additionally, we constructed a phylogenetic tree based on plastome and *ITS* sequences, which revealed that the new species clusters within the *Gigantochloa* clade.

## Graphical abstract

We describe *Gigantochloa falcihumeris*, a new species of paleotropical woody bamboo discovered in Southwest Yunnan, China. It is distinguished by its culm leaf sheath shoulders that prominently rise into a distinctive ca. 1 cm long falcate point. Phylogenetic analyses based on plastome and ITS sequences confirm its placement within the genus *Gigantochloa*.

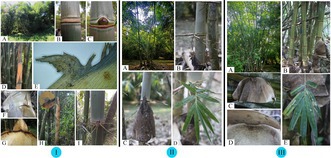

## 1 Introduction

In 1864, *Gigantochloa* was named by Kurz without morphological description (Kurz 1864). It was not until 1868 that Munro provided the first morphological description of the genus in his “Monograph of the Bambusacea” (Munro 1868), marking the formal publication of *Gigantochloa*; however, he did not designate a type species for the genus. In 1956, Holttum conducted a systematic study of *Gigantochloa* and formally designated *G. atter* as the type species of the genus (Holttum 1956), thereby fully establishing *Gigantochloa* as a taxonomic unit.

The typical morphological characteristics of *Gigantochloa* include: Culms erect, sometimes scrambling; internodes setose, basal internodes with light yellow‐white stripes; sheath scars prominent; culm nodes flat. Branches many, dominant branches conspicuous. Culm leaves deciduous, densely setose abaxially; auricles inconspicuous; ligules conspicuous; blades erect or reflexed. Foliage leaves many per branchlet; auricles absent; ligules conspicuous; blades medium to large (Keng and Wang 1996; Li et al. 2006; Yi et al. 2008, 2017). Currently, approximately 63 species of *Gigantochloa* have been recognized (Vorontsova et al. 2017), which are mainly distributed in the rainforests from Southeast Asia to South Asia (Keng and Wang 1996, Li et al. 2006, Yi et al. 2008, 2017). Among these, 9 species have been recorded in China, with seven species found in Yunnan Province (Yi et al. 2017). In recent years, new species of *Gigantochloa* continue to be discovered and published in the Yunnan region (Xu et al. 2021; Qin et al. 2023).

*Gigantochloa* is a paleotropical woody bamboo and is closely related to *Bambusa* Schreber and *Dendrocalamus* Nees (Liu et al. 2020). *Bambusa* can be readily distinguished from the other two genera by vegetative characteristics such as culm leaf blade erect, auricle conspicuous, and having branch thorn. In contrast, *Dendrocalamus* and *Gigantochloa* are highly similar in morphology, both exhibit culm leaf auricle inconspicuous, ligule well‐developed, and blade erect or reflexed (Keng and Wang 1996; Li et al. 2006; Yi et al. 2008, 2017). Except for certain *Gigantochloa* species that can be identified by yellow stripes on internodes, accurate identification generally relies on reproductive organs such as flowers and fruits. However, given the rarity of bamboo flowering, molecular systematics has become an essential supplementary approach in taxonomic studies of these groups (Triplett 2023; Wang et al. 2023; Zhang, Zhang, et al. 2024; Chen et al. 2025a; Li et al. 2025).

In 2022, during a bamboo resource survey in Lincang City, Yunnan Province, we discovered a bamboo with distinctive morphological characteristics; it exhibits 1 or without dominant branch, culm leaf auricle absent, ligule prominent, blade erect, and without branch thorn. These characteristics align with the morphological definitions of both *Gigantochloa* and *Dendrocalamus*. However, its culm leaf sheath shoulder is prominent upward, forming a ca. 1 cm long falcate appendage, which is highly distinctive. As no inflorescence specimens were collected, it was difficult to determine its taxonomic status based solely on vegetative organs. To address this, we collected fresh and healthy leaf samples for total genomic DNA extraction and constructed a phylogenetic tree to clarify its systematic position. The results showed that it clusters within the *Gigantochloa* clade with strong statistical support, leading us to identify it as a new species of *Gigantochloa*, which is hereby formally described.

## 2 Materials and Methods

### 2.1 Field Work, Specimen Collection, and Morphological Comparison

Observations and measurements of living plants were conducted in natural habitat. Specimens were examined under a stereomicroscope (AOSV HD206), while fresh and healthy leaves were collected and dried in silica gel for molecular analyses. The morphological characteristics of closely related species were primarily referenced from the authoritative literature monographs (Keng and Wang 1996; Li et al. 2006; Yi et al. 2008, 2017) and newly described species (Xu et al. 2021). Additionally, specimens deposited in the Herbarium of Southwest Forestry University (SWFC), Herbarium of Kunming Institute of Botany, Chinese Academy of Sciences (KUN), and Herbarium of Xishuangbanna Tropical Botanical Garden, Chinese Academy of Sciences (HITBC), among other herbaria, were examined; at the same time, we also conducted field observations on species of *Gigantochloa* in the Bamboo Garden of Xishuangbanna Tropical Botanical Garden, Chinese Academy of Sciences.

### 2.2 DNA Extraction, Sequencing, and Assembly

Total genomic DNA was extracted from silica gel‐dried leaves using the TIANGEN Magnetic Plant Genomic DNA Kit (TIANGEN, Beijing, China). DNA quality was assessed for degradation, contamination, and concentration by the Agilent 5400 system. Qualified DNA was sheared into approximately 350 bp fragments with a Covaris focused ultrasonicator, followed by end polishing, A‐tailing, ligation of full‐length Illumina sequencing adapters, and PCR amplification. PCR products were accurately quantified using real‐time PCR (3 nM) to determine the effective library concentration. Eligible libraries were sequenced on the Illumina platform with PE150 strategy, generating approximately 2 GB of raw data per sample. All the aforementioned sequencing experiments were conducted at Novogene Bioinformatics Technology Co. Ltd. in Beijing, China.

The raw data were quality‐controlled using Fastp 0.19.7 (Chen et al. 2018) to remove low‐quality sequences. Plastome and nuclear ribosomal DNA (nrDNA) sequence of the new species were assembled using GetOrganelle (Jin et al. 2020) with default parameters. Subsequently, the plastome sequence was visualized using Bandage (Wick et al. 2015), and the assembled sequence was analyzed for collinearity using Mauve (Darling et al. 2004). Finally, Plastome sequence was annotated using CPGAVAS2 (Shi et al. 2019), and annotations were manually adjusted in Geneious Prime 2022.0.1 (Kearse et al. 2012) based on the annotation information of *Dendrocalamus strictus* (Roxb.) Nees (NCBI: MK679802). The nrDNA sequences of the new species were assembled and annotated in Geneious Prime 2022.0.1 (Kearse et al. 2012) with reference to *D. strictus* (Roxb.) Nees (NCBI: JX139103). *ITS* sequences of the new species were accurately extracted using ITSx (Bengtsson‐Palme et al. 2013).

### 2.3 Construct Phylogenetic Tree

Phylogenetic trees of the new species were reconstructed using both Maximum Likelihood (ML) and Bayesian inference (BI) based on plastome and *ITS* sequences. Sequences of related species were retrieved from the NCBI database, comprising a total of 46 sequences from 40 species (Table 1). Sequence alignment was performed using MAFFT (Katoh et al. 2002). For ML analyses, optimal nucleotide substitution models were identified using ModelFinder (Kalyaanamoorthy et al. 2017) based on the Bayesian Information Criterion (BIC). Specifically, K3Pu + F + R4 and TPM2u + G4 were determined as the best‐fit models for plastome and ITS datasets, respectively. ML analysis was conducted with IQ‐TREE 2.2.5 (Nguyen et al. 2015), with branch support assessed through 1000 ultrafast bootstrap replicates and SH‐aLRT tests (Hong et al. 2022). BI was carried out in MrBayes 3.2.7a (Ronquist et al. 2012) under the GTR + I + G model, which was selected via BIC using jModelTest 2.1.7 (Darriba et al. 2012). Two independent Markov Chain Monte Carlo (MCMC) runs were performed for 10,000,000 generations, sampling every 1000 generations. The first 25% of samples were discarded as burn‐in, and a 50% majority‐rule consensus tree was generated when the average standard deviation of split frequencies dropped below 0.01. Nodal support was evaluated based on posterior probabilities (Ronquist et al. 2012).

**TABLE 1:** Voucher information and GenBank accession numbers for plant materials used in this study.

Number	Taxon	Plastome	ITS
1	*Bambusa bambos*	KJ870988	DQ915808
2	*Bambusa beecheyana*	—	AY839720
3	*Bambusa chungii*	—	AY839709
4	*Bambusa emeiensis*	ON969348	AY839711
5	*Bambusa grandis*	NC068816	AY839721
6	*Bambusa intermedia*	MW463057	AY839718
7	*Bambusa membranacea*	—	AY839704
8	*Bambusa multiplex*	OM687226	AY839710
9	*Bambusa nutans*	—	AY839706
10	*Bambusa oldhamii*	NC012927	AY839707
11	*Bambusa sinospinosa*	NC050781	AY839714
12	*Bambusa subaequalis*	—	AY839712
13	*Bambusa surrecta*	—	AY839715
14	*Bambusa textilis*	NC071780	AY839717
15	*Bambusa tuldoides*	PQ014193	AY839708
16	*Bambusa valida*	—	AY839716
17	*Bambusa ventricosa*	MH410121	MH428851
18	*Dendrocalamus asper*	—	KY296047
19	*Dendrocalamus barbatus*	MK679769	MH792021
20	*Dendrocalamus brandisii*	MK679786	DQ270132
21	*Dendrocalamus giganteus*	—	MH271068
22	*Dendrocalamus hamiltonii*	MK679767	KY296051
23	*Dendrocalamus membranaceus*	NC050766	—
24	*Dendrocalamus sinicus*	MK962316	DQ270135
25	*Dendrocalamus* sp.	—	KY296046
26	*Dendrocalamus strictus*	MK679802	JX139103
27	*Dendrocalamus yunnanicus*	MK679784	MH792011
28	*Gigantochloa albociliata*1	NC050765	MH921474
29	*Gigantochloa albociliata*2	—	DQ270130
30	*Gigantochloa atroviolacea*1	MK679803	EU543214
31	*Gigantochloa atroviolacea*2	NC050777	—
32	*Gigantochloa falcihumeris*	PX427808	PX424343
33	*Gigantochloa glabrata*	MK679788	—
34	*Gigantochloa levis*	—	GQ464820
35	*Gigantochloa nigrociliata*1	NC050778	—
36	*Gigantochloa nigrociliata*2	MK679804	—
37	*Gigantochloa parviflora*1	MK679771	JX139104
38	*Gigantochloa parviflora*2	NC050749	—
39	*Gigantochloa verticillata* 1	MN688203	DQ270131
40	*Gigantochloa verticillata* 2	NC050779	—
41	*Gigantochloa verticillata* 3	MK679805	—
42	*Melocalamus arrectus*	MK679766	DQ131518
43	*Melocalamus scandens*	PV976839	DQ131516
*Outgroup*			
1	*Arundinaria fargesii*	MZ905456	HQ292268
2	*Arundinaria qingchengshanensis*	—	HQ292270
3	*Arundinaria gigantea*	JX235347	—

## 3 Results

### 3.1 Morphological Comparisons

*Gigantochloa falcihumeris* is morphologically similar to *G. verticillata* and *G. felix*, but can be clearly distinguished by differences in key morphological characters such as the taller culms, internodes without stripes, culm leaf sheath shoulders prominent upward forming ca. 1 cm long falcate appendage, culm blade erect, culm leaf auricle absent, and culm leaf ligule ca. 5 mm tall (Table 2).

**TABLE 2:** Morphological comparison between *Gigantochloa falcihumeris* and related species.

Characters	*G. falcihumeris*	*G. verticillata*	*G. felix*
Culms	Erect, 7.5–12 (18) cm diameter, 18–23 m tall; apex drooping	Erect, 7–10 cm diameter, 8–15 m tall; apex drooping and inclining	Nearly erect, 4–6 cm in diameter, 9–13 m tall; apex drooping
Internode	Without stripes	Light yellow stripes	Without stripes
Culms leaf sheath	Black hairs abaxially, shoulders prominent upward forming falcate appendage	White and brown setae abaxially, shoulders not prominent	Brown black setae abaxially, shoulders not prominent
Culm blade	Erect	Reflexed	Erect
Culm leaf auricle	Absent	Inconspicuous	Absent
Culm leaf ligule	ca. 5 mm tall, margins dentate	ca. 3 mm tall, margins fimbriate or dentate	ca. 1 mm tall, margin serrulate
Foliage leaf	14–26 cm × 2.5–4 cm, both surface slightly scabrous	24–47 cm × 3.5–7 cm, both surface glabrous	30–45 cm × 4.5–6 cm, pubescent abaxially
Foliage leaf ligule	1–2 mm tall, margin serrulate	5–10 mm tall, margins entire	3–4 mm tall, margins bifid or concave
Transverse veins	Inconspicuous	Conspicuous	Inconspicuous

### 3.2 Phylogenetic Analysis

**FIGURE 1:**
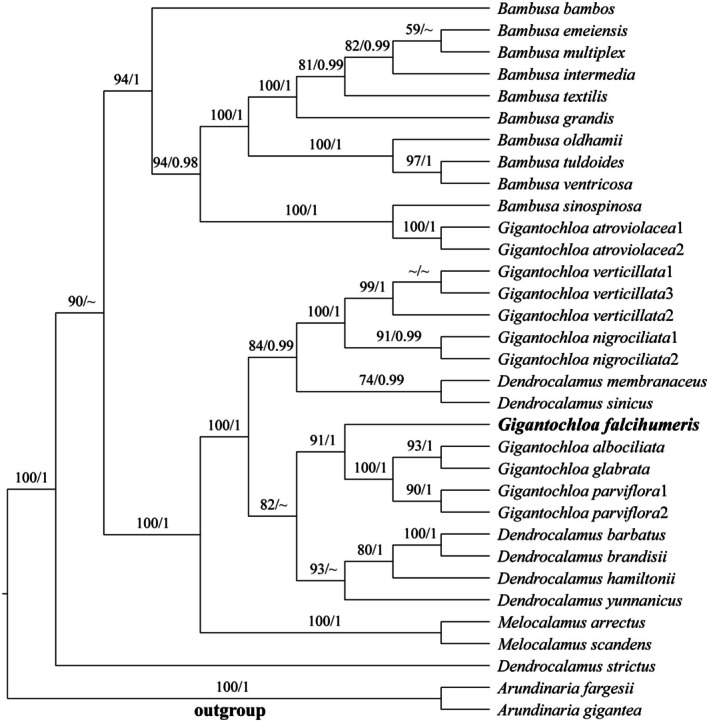
Phylogenetic tree reconstructed by Maximum Likelihood (ML) analysis based on plastome sequences. Numbers along branches indicate the Maximum Likelihood bootstrap values (MLBP) (left) and Bayesian posterior probabilities (BI) (right). “~”: nodes with MLBP < 50% (left), Bayesian posterior probabilities (BI) < 95% (right).

Upon alignment, the total length of the plastome sequence was 102,776 bp, including 2017 variable sites and 1384 parsimony information sites. In the plastome phylogenetic tree, the clustering of various species was relatively poor: two sequences of *Gigantochloa atrovilacea* were nested within the *Bambusa* clade, while *Dendrocalamus membranaceus* and *D. sinicus* were grouped with *Gigantochloa* species, and other *D. strictus* formed a distinct monophyletic clade (Figure 1). Despite the relatively disorganized phylogenetic relationships among species in the plastome tree, the new species within a clade containing species of *Gigantochloa* (MLBP/BI = 91/1) was most closely related to *G. albociliata*, *G. glabrata* and *G. parviflora* (Figure 1).

**FIGURE 2:**
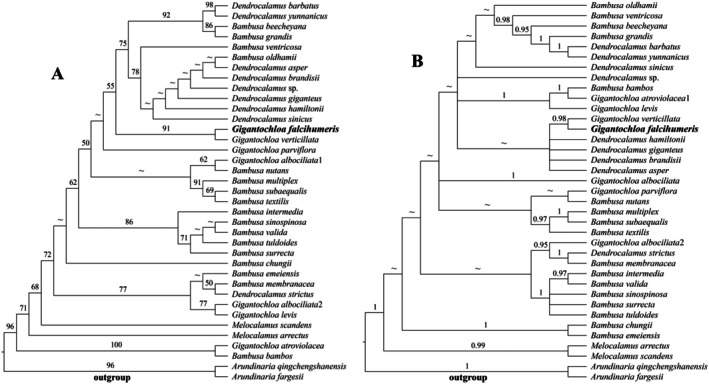
Phylogenetic tree reconstructed from *ITS* sequences. (A) Maximum Likelihood (ML) method. (B) Bayesian Inference (BI) method. Numbers along branches indicate the Maximum Likelihood bootstrap values (MLBP) (A) and Bayesian posterior probabilities (BI) (B). “~”: nodes with MLBP < 50% (A), Bayesian posterior probabilities (BI) < 95% (B).

The total length of the *ITS* sequences was 285 bp, containing 182 variable sites and 104 parsimony‐informative sites. In the *ITS* phylogenetic tree, the interspecific evolutionary relationships were more disorganized than those in the plastome tree. Additionally, the topologies generated by ML and BI methods showed inconsistencies; however, both methods consistently grouped the new species with *G. verticillata* on the same clade with strong support (MLBP = 91, BI = 0.98) (Figure 2).

## 4 Taxonomy

**FIGURE 3:**
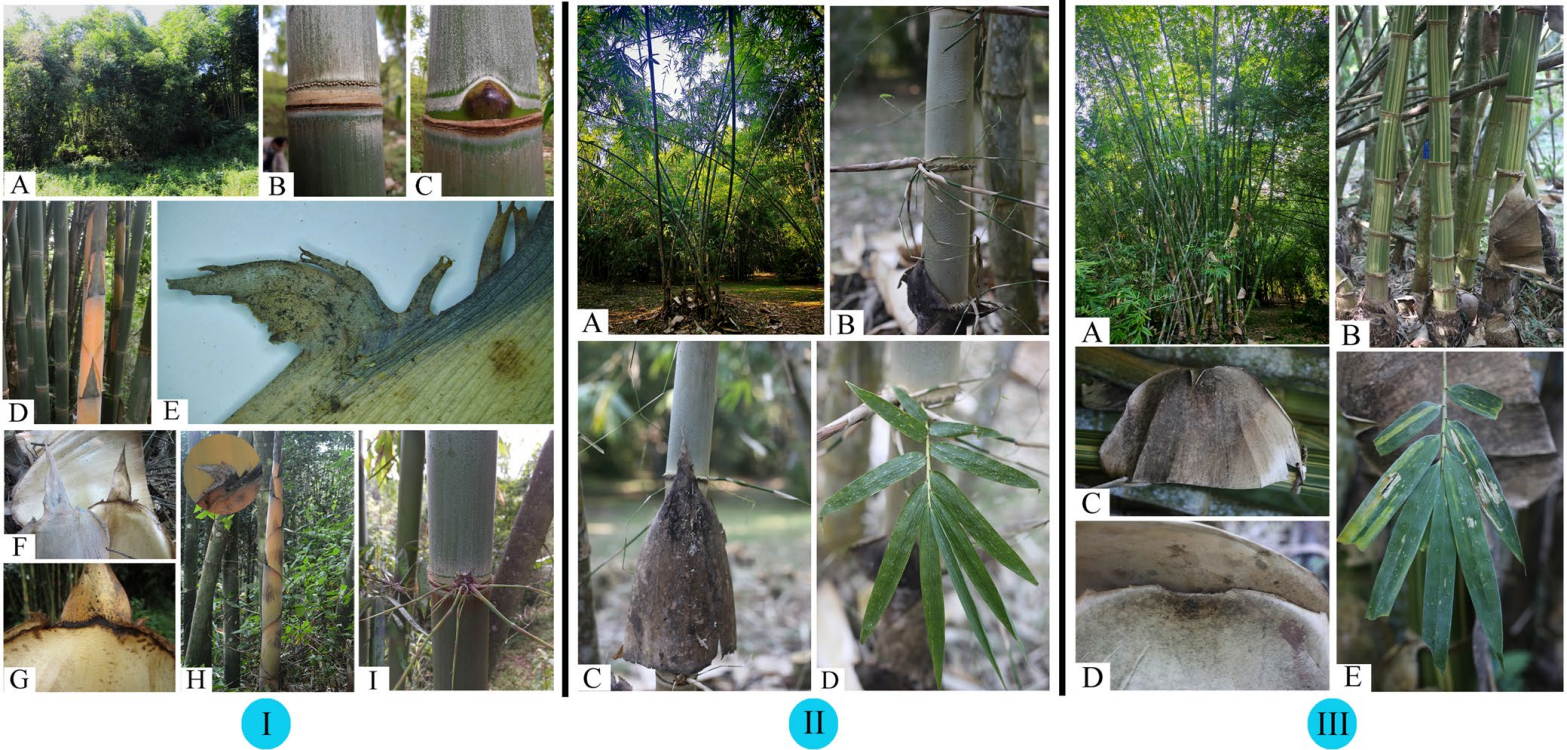
(I) *Gigantochloa falcihumeris*: (A) Habitat, (B) culm fragment showing nodal line, (C) culm bud, (D) new shoot showing culm leaves, (E) falcate appendage, (F) culm leaf sheath shoulders prominent, (G) culm leaf ligule, (H) bamboo shoot and falcate appendage, (I) branch complement. (II) *Gigantochloa felix*: (A) clump, (B) branch complement, (C) culm leaves, (D) foliage leaves. (III) *Gigantochloa verticillata* : (A) clump, (B) internodes yellow striate, (C) culm leaves, (D) culm leaf ligule, (E) foliage leaves.

**FIGURE 4:**
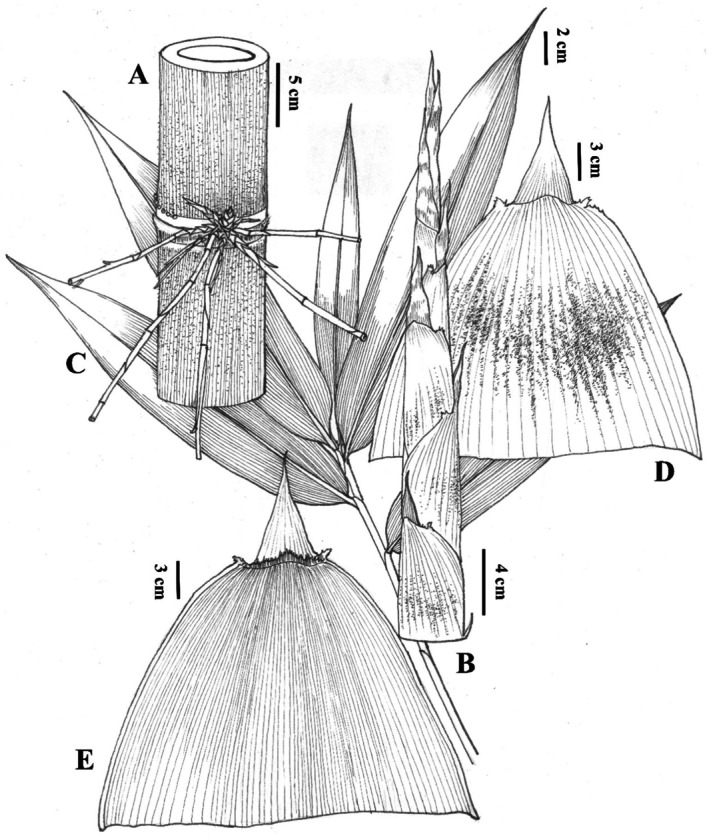
*Gigantochloa falcihumeris*: (A) Branch complement, (B) bamboo shoot, (C) foliage leaf, (D) culm leaf, adaxial view, (E) culm leaf, abaxial view.

*Gigantochloa falcihumeris* C.M.Hui & J.W.Li sp. nov. (Figures 3I and 4).

### 4.1 Type

CHINA. Yunnan: Lincang City, Cangyuan County, Menglai Township, Papeng Village, in sparse forests, N23°11′ 57.40″, E99°14′ 52.33″, elev. ca. 1380 m, 24 September 2024, *C. M. Hui, J. W. Li, H. F. Bao & C. H. Zhang 0072417*(holotype: SWFC!).

### 4.2 Diagnosis

*Gigantochloa falcihumeris* is morphologically similar to *G. verticillata* and *G. felix*, but can be distinguished by the following characteristics: Culms 7.5–12 (18) cm in diameter, 18–23 m tall (vs. 7–10 cm in diameter, 8–15 m tall in *G. verticillata* ; 4–6 cm in diameter, 9–13 m tall in *G*. *felix*); internodes without stripes (vs. light yellow stripes in *G. verticillata* ); culm leaf sheath shoulders prominent upward forming ca. 1 cm long falcate point (vs. shoulders not prominent in *G. verticillata* and *G. felix*); culm blade erect (vs. reflexed in *G. verticillata* ); culm leaf auricle absent (vs. inconspicuous in *G. verticillata* ); culm leaf ligule ca. 5 mm tall (vs. ca. 3 mm tall in *G. verticillata* ; ca. 1 mm tall in *G. felix*) (Table 2).

### 4.3 Description

Rhizomes simpodial. Culms erect, the apex drooping, 7.5–12 (18) cm in diameter, 18–23 m tall; internodes 30–38 (40) cm long, without stripes, initially covered with white hairs; intranode ca. 1 cm tall, with a ring of white tomentum and aerial roots; culm nodes flat, glabrous; sheath scar slightly prominent with dense brown setae. Branch developing from higher nodes; branches several with 1 or without dominant branch. Culm leaves deciduous, leathery; sheaths 28–35 cm × 33–42 cm, equal to or slightly shorter than internodes, abaxially white powdery and covered with deciduous black hairs initially, mid‐upper with a ca. 5 mm wide thin marginal membrane, shoulders prominent upward forming ca. 1 cm long falcate appendage with margins serrate; blades erect, 8–12 cm long, rugose, sparsely white powdery, margins involute; auricle absent; ligule ca. 5 mm tall, margins dentate. Foliage leaves 6–10 per ultimate branch; blade elliptic‐lanceolate, thin‐textured, 14–26 cm × 2.5–4 cm, both surface slightly scabrous, secondary veins 8–12 pairs, transverse veinlets inconspicuous; pseudopetiole 3–5 mm long, glabrous; sheath 4–9 cm long, covered with procumbent black hairs; auricle absent; ligule 1–2 mm tall, glabrous, margin finely dentate. Inflorescence and caryopsis unknown.

### 4.4 Phenology

New shoots June to September.

### 4.5 Distribution and Habitat

**FIGURE 5:**
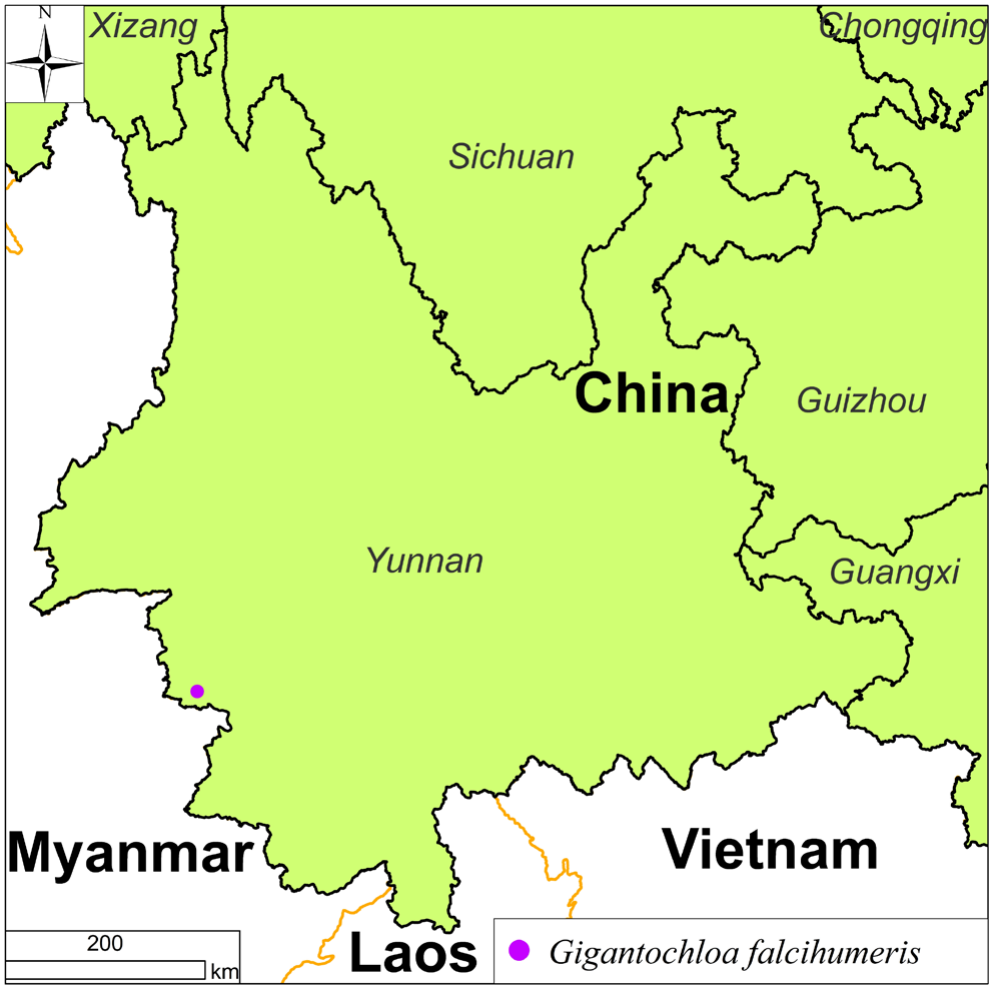
Geographical distribution of *Gigantochloa falcihumeris* C.M.Hui & J.Wei Li.

*Gigantochloa falcihumeris* was recently found near Papeng Village at an elevation of 1200–1400 m in Menglai Township, Cangyuan County, Southwestern Yunnan, China (Figure 5). It coexists with other species, such as *Lithocarpus fenestratus* (Roxb.) Rehder, *Castanopsis indica* (Roxb. ex Lindl.) A. DC., *Dendrocalamus sinicus* L.C. Chia & J.L. Sun, *D. brandisii* (Munro) Kurz, and *Castanopsis hystrix* Hook. f. & Thomson ex A. DC.

### 4.6 Etymology

The specific epithet “*falcihumeris*” refers to the culms leaf sheath shoulders prominently upward forming falcate appendage.

### 4.7 Chinese Name

zhí gǎn jù zhú (Chinese pronunciation); 直杆巨竹(Chinese name); yě duō wǎ (local Lahu language).

### 4.8 Local Use

The local Lahu people refer to the new species as “yě duō wǎ” (meaning “rafter bamboo”). Due to its straight culms and moderate size, it has traditionally been used as rafters for building houses. Additionally, the local Lahu people also use its bamboo shoots to make pickled sour bamboo shoots.

### 4.9 Conservation Status

*Gigantochloa falcihumeris* is so far only known from its type locality, with a population of approximately five clumps, and the number of mature individuals in other similar habitats remains unknown. It is threatened by potential habitat disturbance and a lack of comprehensive surveys to define its full distribution. It can be categorized as “Data Deficient” (DD) following the IUCN categories and criteria (IUCN 2024).

### 4.10 Additional Specimens Examined

CHINA. Yunnan: Lincang City, Cangyuan County, Menglai Township, Papeng Village, in sparse forests, N23°11′ 57.40″, E99°14′ 52.33″, elev. ca. 1380 m, 24 September 2024, *C. M. Hui, J. W. Li, H. F. Bao & C. H. Zhang*, *0072418*, *0072419*, *0072420*(SWFC!).

## 5 Discussion

Due to the exceptionally long flowering cycles of bamboo plants (Zheng et al. 2020; Chen et al. 2025b), their classification and identification primarily rely on morphological characteristics of vegetative organs (such as culms, culm sheaths, and foliage leaves). This approach has been commonly used for establishing new bamboo species in traditional taxonomy (Yi 1992, 2001; Wen 2001; Du et al. 2013; Zeng et al. 2014; Chen et al. 2013; Zhang et al. 2019; Ye et al. 2020, 2021; Shi et al. 2024; Zhang, Cao, and Ding 2024). In recent years, the integration of molecular phylogenetic data has provided further critical evidence for delineating new bamboo species (Triplett 2023; Wang et al. 2023; Zhang, Zhang, et al. 2024; Chen et al. 2025a; Li et al. 2025).

Through morphological comparison and phylogenetic reconstruction, this study identifies an unknown bamboo species as a new member of *Gigantochloa*. However, we constructed phylogenetic trees based on plastome and ITS sequences respectively, and found that the evolutionary relationships revealed by different datasets were significantly different. Specifically, the ML tree and BI tree constructed from plastome data exhibited consistent topological structures; in contrast, the phylogenetic trees based on ITS data not only showed inconsistent topologies but also had more complex branching relationships than the plastome trees. Nevertheless, both types of phylogenetic trees placed the new species within the *Gigantochloa* clade. Previous studies have generally adopted the approach of constructing trees using multiple datasets separately to comprehensively determine the systematic position when establishing new bamboo species (Zhang, Zhang, et al. 2024; Chen et al. 2025b; Li et al. 2025). This indicates that judging the taxonomic affiliation of a new species solely based on a phylogenetic tree constructed from a single dataset is insufficient and may lead to one‐sided conclusions.

Furthermore, this study also found that neither the phylogenetic trees constructed based on plastome nor ITS sequences exhibited a clear and stable evolutionary relationship structure at the species level. This phylogenetic ambiguity is likely related to the “*Bambusa–Dendrocalamus–Gigantochloa* (BDG) complex”—a group primarily composed of genera such as *Bambusa*, *Dendrocalamus*, *Gigantochloa*, and *Melocalamus* (Zhou et al. 2017). This complex represents the most diverse and phylogenetically challenging group within the paleotropical woody bamboos (PWB) of the grass subfamily Bambusoideae and has long presented a major challenge in bamboo systematics research (Sun et al. 2005; Yang et al. 2010; Goh et al. 2013; Zhou et al. 2017; Liu et al. 2020, 2024). Although significant progress has been made in recent studies (Liu et al. 2020, 2024), the intergeneric boundaries and evolutionary relationships within this complex remain not yet fully resolved.

According to records from *Flora Reipublicae Popularis Sinicae* (1996), *Flora of China* (2006), *Iconographia Bambusoidearum Sinicarum* (2008), and *Illustrated Flora of Bambusoideae in China* (2017), species of *Gigantochloa* in China are primarily distributed in the Xishuangbanna Prefecture of southern Yunnan, with very sporadic records in Dehong Prefecture. The new species discovered in Cangyuan County in southwestern Yunnan, which not only adds a new species to *Gigantochloa* but also expands the known geographic distribution range of *Gigantochloa* in China. Situated on the southwestern border of China and adjacent to Myanmar, Cangyuan County exhibits strong Southeast Asian floristic characteristics in its plant vegetation. However, due to its remote location and the constraints of local economic development, biodiversity baseline surveys remain relatively limited, resulting in many species yet to be documented. Enhanced field investigations in this region should be conducted in the future to further enrich the understanding of both Chinese and global biodiversity.

## Author Contributions

**Jian‐Wei Li:** conceptualization (lead), formal analysis (lead), investigation (lead), writing – original draft (lead). **Chao‐Mao Hui:** data curation (equal), formal analysis (lead), funding acquisition (lead), methodology (equal), project administration (equal), resources (lead), supervision (equal), writing – review and editing (lead). **Wei‐Yi Liu:** conceptualization (equal), methodology (equal), supervision (equal), writing – review and editing (equal). **Mao‐Sheng Sun:** formal analysis (equal), methodology (equal). **Wan‐ling Qin:** investigation (equal). **Hao‐Feng Bao:** investigation (equal). **Ru‐li Zhang:** methodology (equal).

## Funding

This work was supported by the National Key Research and Development Project of China (2021YFD2200501), the Agriculture Joint Special Project of Yunnan Province (202301BD070001‐123), the National‐level high‐quality variety “Yuntian No. 1” high‐standard cultivation technology promotion demonstration (YUN [2024]TG 11 No), the Monitoring Project of Bamboo Forest Ecosystem Positioning Observation and Research Station in South Yunnan, China (2025–YN–15), and the Sci‐Tech Service Station of Farmer Academician in Lancang and Changning City.

## Conflicts of Interest

The authors declare no conflicts of interest.

## Data Availability

All of the data that support the findings of this study are available in the main text.
